# Noradrenalin and dopamine receptors both control cAMP-PKA signaling throughout the cerebral cortex

**DOI:** 10.3389/fncel.2014.00247

**Published:** 2014-08-21

**Authors:** Shinobu Nomura, Maud Bouhadana, Carole Morel, Philippe Faure, Bruno Cauli, Bertrand Lambolez, Régine Hepp

**Affiliations:** ^1^Sorbonne Universités, UPMC Université Paris 06, UM CR 18, Neuroscience Paris SeineParis, France; ^2^Centre National de la Recherche Scientifique (CNRS), UMR 8246Paris, France; ^3^Institut National de la Santé et de la Recherche Médicale (INSERM), U 1130Paris, France

**Keywords:** catecholamines, GPCR, protein kinase A, imaging, cerebral cortex

## Abstract

Noradrenergic fibers innervate the entire cerebral cortex, whereas the cortical distribution of dopaminergic fibers is more restricted. However, the relative functional impact of noradrenalin and dopamine receptors in various cortical regions is largely unknown. Using a specific genetic label, we first confirmed that noradrenergic fibers innervate the entire cortex whereas dopaminergic fibers were present in all layers of restricted medial and lateral areas but only in deep layers of other areas. Imaging of a genetically encoded sensor revealed that noradrenalin and dopamine widely activate PKA in cortical pyramidal neurons of frontal, parietal and occipital regions with scarce dopaminergic fibers. Responses to noradrenalin had higher amplitude, velocity and occurred at more than 10-fold lower dose than those elicited by dopamine, whose amplitude and velocity increased along the antero-posterior axis. The pharmacology of these responses was consistent with the involvement of Gs-coupled beta1 adrenergic and D1/D5 dopaminergic receptors, but the inhibition of both noradrenalin and dopamine responses by beta adrenergic antagonists was suggestive of the existence of beta1-D1/D5 heteromeric receptors. Responses also involved Gi-coupled alpha2 adrenergic and D2-like dopaminergic receptors that markedly reduced their amplitude and velocity and contributed to their cell-to-cell heterogeneity. Our results reveal that noradrenalin and dopamine receptors both control cAMP-PKA signaling throughout the cerebral cortex with moderate regional and laminar differences. These receptors can thus mediate widespread effects of both catecholamines, which are reportedly co-released by cortical noradrenergic fibers beyond the territory of dopaminergic fibers.

## INTRODUCTION

The catecholamines dopamine (DA) and noradrenalin (NA) are neurotransmitters that widely modulate brain circuits and behaviors. NA is involved in arousal, attention, memory, and stress whereas DA is implicated in learning, reward, attention, and movement control. Likewise, catecholaminergic dysfunctions are associated with cognitive, emotional, and motor disorders and catecholaminergic transmission is the target of multiple drugs used in therapy of human brain disorders. Catecholamines are synthesized in discrete brainstem nuclei via a common pathway involving tyrosine hydroxylase (TH) that leads to DA production, which is converted to NA by DA beta hydroxylase (DBH). The effects of DA and NA are mediated by G protein-coupled receptors. The five DA receptors belong to the D1/D5 or D2-like classes, which activate or inhibit cAMP/protein kinase A (PKA) signaling via Gs or Gi proteins, respectively (Beaulieu and Gainetdinov, 2011). NA also regulates the cAMP/PKA pathway by activating Gs-coupled beta1–3 receptors or Gi-coupled alpha2 adrenoceptors. NA additionally activates the phospholipase C pathway through Gq-coupled alpha1 receptors (Bylund, 1992; Cotecchia, 2010; Evans et al., 2010).

Dopamine- and NA-containing fibers exhibit wide, but distinctive, distributions in the brain. Catecholaminergic projections to the cerebral cortex stem from DA neurons of the ventral tegmental area (VTA) and NA neurons of the locus coeruleus (LC). LC fibers innervate the entire cortical mantle, whereas VTA fibers distribute in all layers of medio-frontal and ventro-lateral cortices, but are restricted to deep layers in other areas of the rodent cortex (Morrison et al., 1978, 1979; Descarries et al., 1987; Berger et al., 1991; Latsari et al., 2002). The broad distribution of NA receptors in the rodent cortex is consistent with that of LC fibers (Nicholas et al., 1996; Venkatesan et al., 1996; Papay et al., 2006; Paschalis et al., 2009). In contrast, a mismatch exists between the widespread expression also reported for DA receptors (Ariano and Sibley, 1994; Khan et al., 1998; Luedtke et al., 1999; Lemberger et al., 2007; Rivera et al., 2008; Oda et al., 2010), and the restricted distribution of VTA fibers in the rodent cortex. While LC fibers are a plausible source of cortical DA outside the VTA projection areas (Devoto et al., 2005, 2008), the question of the relative functional impact of DA and NA receptors in various cortical regions has not been addressed.

In the present study, we first examined the distribution of LC and VTA fibers in the rodent cortex using site and cell-type-specific labeling of catecholaminergic neurons with green fluorescent protein (GFP) via conditional viral transfer. We then characterized the functional impact of Gs- and Gi-coupled DA and NA receptors on cAMP/PKA signaling in layers II/III and V of the frontal, parietal, and occipital cortex using 2-photon imaging of a genetically encoded PKA sensor in rat brain slices (Bonnot et al., 2014). Our results confirm the differential distribution of LC and VTA fibers in the cortex and reveal that both NA and DA receptors control cAMP-PKA signaling throughout the cerebral cortex with moderate regional and laminar differences.

## MATERIALS AND METHODS

### ANIMALS

All the experiments were performed according to the guidelines of the French Ministry of Agriculture, Food Processing Industry and Forestry for handling animals (decree 2013-118). Transgenic DBH-Cre mice (DBH-cre) were a gift from Bruno Giros [McGill University, Canada, MMRRC line: Tg(Dbh-cre) KH212Gsat/Mmucd, stock number 032081-UCD (Gong et al., 2007)]. DA transporter-Cre mice (DAT-cre) were a gift from Uwe Maskos [Institut Pasteur, France, (Tg)BAC-DATiCrefto (Turiault et al., 2007)]. Male Wistar rats (12–15 days old) were obtained from Janvier Labs. Animals were maintained in a 12 h light–12 h dark cycle, in stable conditions of temperature (22°C), with food and water available *ad libitum*.

### AAV PRODUCTION AND STEREOTACTIC INJECTION

Site- and cell-type-specific labeling of NA or DA neurons was achieved by stereotactic injection of a viral vector into the LC of DBH-Cre or the VTA of DAT-Cre mice (1–3 month-old). The viral vector was a recombinant adeno-associated virus (AAV) driving Cre-dependent expression of a fusion protein containing channelrhodopsin 2 (ChR2) and a yellow variant (YFP) of the GFP from *Aequorea victoria*. The Cre-inducible vector AAV2/1-EF1a-DIO-hChR2(H134R)-EYFP-WPRE-HGHpA (titer: 3 × 10^11^ gc/ml) was produced from Addgene plasmid #20298 at the vector core facility of Nantes University (UMR 1089 IRT1, France). Aliquots of the pseudovirion were stored at -80°C before stereotactic injection. Mice (six DAT-Cre and six DBH-Cre) were anesthetized with isoflurane and placed on a small animal stereotactic frame. For transduction of LC NA neurons, AAV-EF1α-DIO-ChR2-YFP pseudovirion was bilaterally injected adjacent to the LC at coordinates from bregma: antero-posterior (AP), -5.45 mm; medio-lateral (ML), ±1 mm; dorso-ventral (DV) -3.65 mm). For DA neuron transduction, pseudovirion was bilaterally injected into the VTA (AP: -3.4 mm, ML: ±0.5 mm, DV: -4.4 mm). Injections were performed through an internal canula at a rate of 0.1 μl/min for 10 min (total 1 μl per site). The canula was held in place for 15 min before retraction out of the brain.

### IMMUNOHISTOCHEMISTRY

Eight weeks after injection, mice were deeply anesthetized with 10 mg/ml ketamine and 0.1% xylazine before transcardiac perfusion with 50 ml of 4% paraformaldehyde in 0.12 mM phosphate buffer (pH 7.4). Brains were removed and post-fixed with the same solution for 2 h, cryoprotected with 30% sucrose and cut with a freezing microtome (Leica) at a thickness of 40 μm. Slices were washed overnight with PBS, then blocked and permeabilized with PBS complemented with 0.2% fish skin gelatin, 0.25% triton X-100 (PBS-GT) for 2 h at room temperature. Brain slices were incubated overnight at 4°C with primary antibody diluted in PBS-GT (monoclonal mouse anti TH: MAB318 (Clone LNC1), Millipore, 1/2000; polyclonal chicken anti-GFP: GFP-1020 (Aves Labs, 1/2000). After 6 ×10 min washes in PBS-GT, slices were incubated for 3 h at room temperature with fluorescently labeled secondary antibody diluted at 1/1000 in PBS-GT (goat anti chicken alexa488, Invitrogen A11039; goat anti mouse Alexa555, Invitrogen A21422). Slices were mounted in fluoromount (Clinisciences) after extensive washes in PBS-GT followed by PBS. Images were obtained using an AXIO Zoom.V16 macroscope (Zeiss). Labeled fibers were drawn in black (value of the pixel: 255) on white background (value of the pixel: 0) using Image J and Photoshop Cs2 (Adobe). The density of fibers was obtained from drawings using the line plot profile tool of ImageJ on lanes of 200 pixels (260 μm) width covering the vertical extent of the cortex from pia to white matter. The fiber density corresponds to the averaged value of black and white pixels in a 200 pixel line projected on the vertical axis along the lane.

### BRAIN SLICE PREPARATION AND VIRAL TRANSDUCTION FOR IMAGING PKA ACTIVITY

Rats were killed by decapitation. Brains were quickly removed and immersed in ice-cold artificial cerebrospinal fluid (ACSF) containing (in mM): 126 NaCl, 2.5 KCl, 1.25 NaH_2_PO_4_, 2 CaCl_2_, 1 MgCl_2_, 26 NaHCO_3_, 20 D-glucose, 5 Na pyruvate, 1 kynurenic acid, and saturated with 5% CO_2_/95% O_2_. Parasagittal slices (300 μm thick) of cortex were cut at an angle of 10° using a Leica VT 1000S Vibratome (Leica). Slices were kept at room temperature for 30 min in the same solution. Brain slices were placed onto a millicell-CM membrane (Millipore) with culture medium (50% minimum essential medium, 50% Hanks’ balanced salt solution, 6.5 g/L glucose, and 100 U/ml penicillin/100 μg/ml streptomycin; Invitrogen). Transduction was performed by adding ∼5 × 10^5^ particles per slice of sindbis virus encoding the GAkdYmut sensor that reports PKA activation through a reversible increase of fluorescence intensity (Bonnot et al., 2014). Slices were incubated overnight at 35°C in 5% CO_2_. The next morning, brain slices were equilibrated in ACSF for 1 h and then placed into the recording chamber and perfused continuously at 2 ml/min with ACSF at 32°C. Under these conditions, sindbis viral transduction efficiently and selectively targets cortical pyramidal neurons, and leaves their functional properties essentially unaffected (Drobac et al., 2010; Hu et al., 2011).

### OPTICAL RECORDINGS

Two-photon images were obtained with a custom-built 2-photon laser scanning microscope as described (Bonnot et al., 2014), based on an Olympus BX51WI upright microscope (Olympus, Tokyo, Japan) with ×40 (0.8 NA) or ×60 (0.9 NA) water-immersion objectives and a titanium:sapphire laser (MaiTai HP; Spectra Physics, Ellicot City, MD, USA). Two-photon excitation was performed at 920 nm for GFP. Images were acquired as z stacks and analyzed using ImageJ (U.S. National Institutes of Health, Bethesda, MD, USA; http://rsbweb.nih.gov/ij/). Occasional *x*, *y*, and *z* drifts were corrected using custom macros developed from ImageJ plugins TurboReg, StackReg (Thevenaz et al., 1998), MultiStackReg, and Image CorrelationJ (Chinga and Syverud, 2007). Fluorescence variations were measured at the soma of pyramidal neurons. Fluorescence intensity of regions of interest (ROIs) was calculated for each time point from average intensity projection of 3–5 frames by averaging pixel intensity. Variations of fluorescence intensity in a given ROI were expressed as the ratio Δ*F*/*F*0 and calculated according to the formula (*F - F*0)/*F*0. *F* corresponds to the fluorescence intensity in the ROI at a given time point, and *F*0 corresponds to the mean fluorescence intensity in the same ROI during control baseline prior to drug application. Pseudocolor hue saturation value (HSV) encoding of fluorescence intensity was performed using IGOR Pro (WaveMetrics) custom procedures. Pseudocolor images were obtained by dividing, pixel-by-pixel a raw fluorescence image *F* by the *F*0 image averaged over several time points prior to drug application. Color coding displays the ratio *F*/*F*0 (in hue) and the fluorescence *F* (in value). The EC_50_ for NA and DA were obtained by fitting the values with the Hill equation using IgorPro6. The same equation was used to determine the 10–90% rise time of the agonist induced PKA activation.

### DRUGS

All drugs were bath applied. SKF38393, SCH23390; Propranolol, Yohimbin, CGP20712, and forskolin were purchased from Tocris. NA, DA, haloperidol, and isoproterenol were from Sigma-Aldrich.

### STATISTICS

In this report, *N* represents the number of animals or brain slices tested while n represents the number of cells on which measurements were performed. Values are expressed as mean ± SEM. Statistical significance was assessed with Student’s *t*-test. *p* value < 0.05 was considered statistically significant.

## RESULTS

### DISTRIBUTION OF LC AND VTA FIBERS IN THE CEREBRAL CORTEX

**FIGURE 1:**
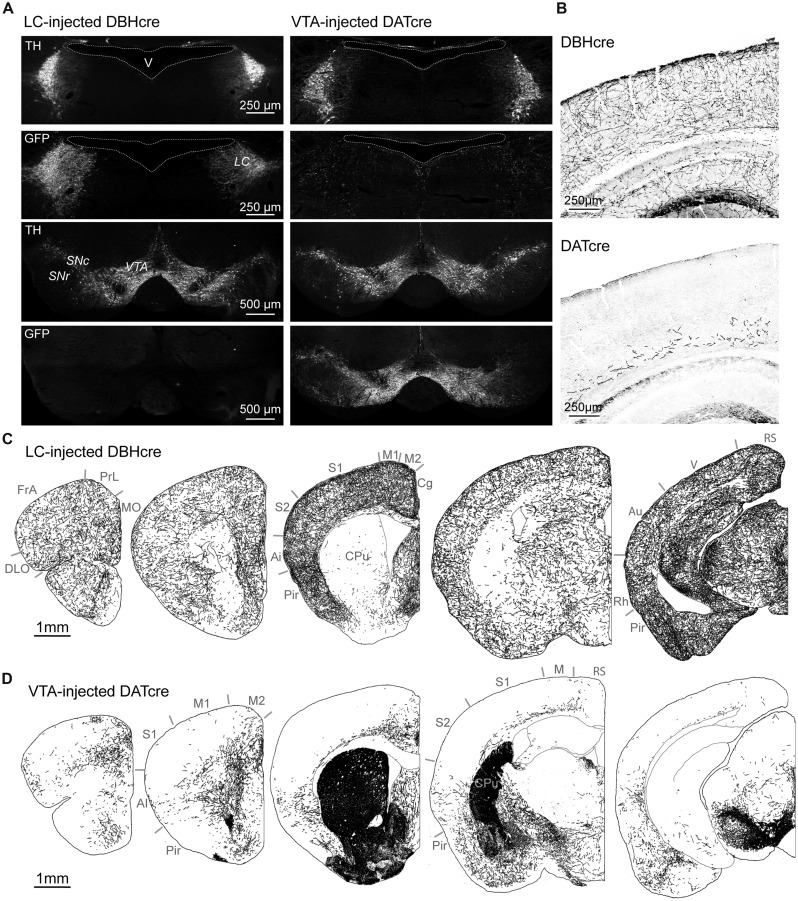
**Catecholaminergic projections to the cortex. (A)** Immuno labeling against TH and GFP in the LC and VTA 8 weeks after bilateral injection of a Cre-dependent AAV expressing GFP in the LC of DBH-Cre mice or the VTA of DAT-Cre mice. Note that GFP labeling in VTA-injected DAT-Cre mice extends to neighbor DA neurons of the substantia nigra pars compacta (SNc) but is absent from NA neurons of the LC. **(B)** Immunolabeling of GFP-expressing fibers in the somato-sensory parietal cortex of DBH-Cre and DAT-Cre mice. **(C,D)** Drawings of NA or DA fibers obtained from immunolabeling of GFP positive fibers in coronal brain sections of LC-injected DBH-Cre and VTA-injected DAT-Cre mice (antero-posterior from left to right). Note the different distribution of NA and DA fibers in cortical areas and caudate-putamen (CPu). Ai: agranular insular cortex; Au: auditory cortex; Cg: cingulate cortex; DLO: dorso-lateral orbital cortex; FrA: frontal association cortex; M1-M2-M: primary or secondary motor cortex; MO: medial orbital cortex; Pir: piriform cortex; PrL: prelimbic cortex; Rh: rhinal cortex; RS: retrosplenial cortex; S1: primary somatosensory cortex; V: visual cortex.

**FIGURE 2:**
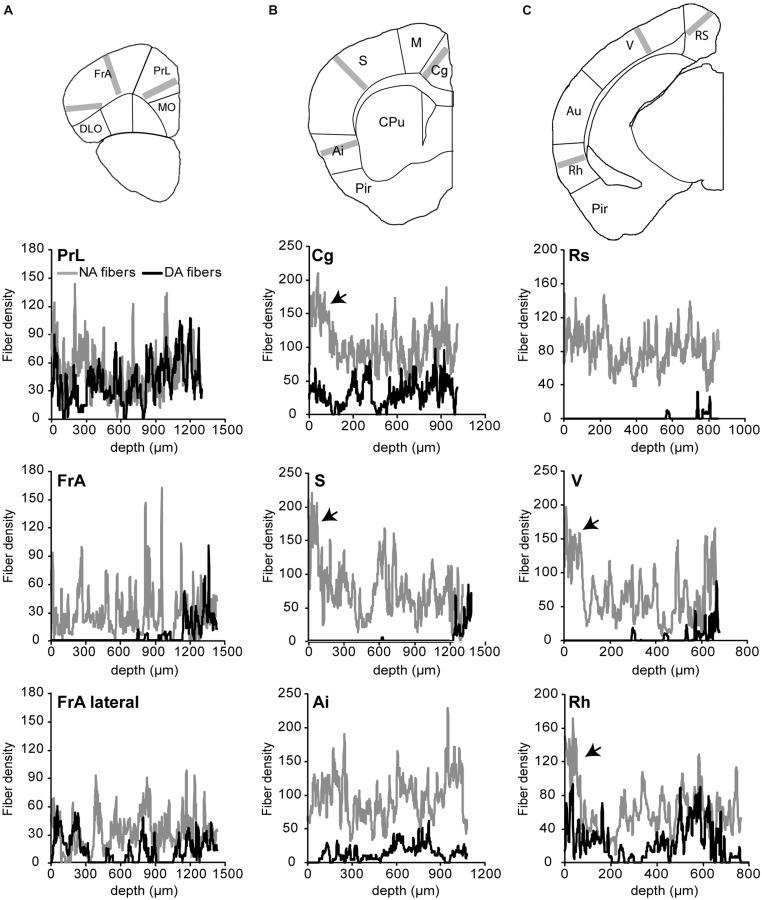
**Density of noradrenergic and dopaminergic fibers in various areas of the cortex.** The density of fibers was obtained from the drawings shown in **Figure 1** on lanes of 200 pixels (260 μm) width orthogonal to the pial surface, as depicted in images of the upper panel (gray bars). The fiber density, measured in three regions of anterior **(A)**, intermediate **(B)**, and posterieor **(C)** coronal brain sections, corresponds to the averaged pixel value along the lane plotted on the x-axis of the graphs from the pial surface to indicated depths. NA fibers are widespread in the cortex and are enriched (arrows) in upper layers of cingulated (Cg), somato-sensory (S), visual (V), and rhinal (Rh) cortices. Note that DA fibers are absent from most layers of a rostro-caudal band comprising the medio-lateral part of the frontal association (FrA), S and V cortices, except for a thin lamina in deep layers.

In order to compare LC and VTA projections to the rodent cortex, we selectively expressed GFP in DA or NA neurons using Cre-dependent viral transduction (see Materials and Methods). DBH-Cre mice (*N* = 6) and DAT-Cre mice (*N* = 6) were injected in the LC or in the VTA, respectively, with a recombinant pseudovirion driving GFP expression at the membrane of Cre-positive neurons. Anti-GFP and anti-TH immunohistochemistry showed that GFP was widely expressed in the LC of DBH-Cre mice and in the VTA and substantia nigra of DAT-Cre mice following viral transduction (**Figure 1A**). In LC-injected DBH-Cre mice, 83.9 ± 2.7% (*n* = 227; *N* = 5) of TH-positive neurons in the LC expressed GFP. Similarly, 86.1 ± 1.2% (*n* = 158; *N* = 3) of TH-positive neurons in the VTA co-expressed GFP. Conversely, LC neurons of VTA-injected DAT-Cre mice and VTA neurons of LC-injected DBH-Cre mice were GFP-negative (**Figure 1A**). These results show that the present protocol of conditional viral transduction enabled efficient site- and cell-type-specific GFP expression in LC NA neurons or VTA DA neurons. We next examined the distribution of NA and DA fibers in the cerebral cortex. GFP labeling revealed a high density of NA fibers throughout all cortical regions and layers examined (**Figures 1B,C**). Quantification of NA fibers (see Materials and Methods) revealed moderate laminar and regional differences, such as an overall lower density in frontal than other regions and a higher density in upper layers in cingulate, somatosensory, rhinal, and visual than in other layers of these regions (**Figure 2**). Similar results were obtained on six LC-injected DBH-Cre mice. DA fibers had a more contrasted distribution. The highest density of fibers was observed in a medial rostro-caudal band that comprised all layers of the prelimbic cortex and extended caudally to the cingulated cortex, but did not reach the retrosplenial occipital area (**Figures 1D** and **2**). A lateral rostro-caudal band of lower fiber density comprised all layers of the lateral part of the frontal association cortex, of the agranular insular cortex and of the rhinal occipital area. Between these two bands, DA fibers were restricted to deep layers in a large region extending from the medial part of the frontal association to the visual cortex through the somatosensory area (**Figures 1D** and **2**). Similar results were obtained on six VTA-injected DAT-Cre mice. The striatum contained a dense network of DA fibers but was sparsely innervated by NA fibers (**Figures 1C,D**), as expected from their known distributions (Aston-Jones, 2004; Gerfen, 2004). These data confirm that NA fibers widely innervate the cortical mantle, whereas the distribution of DA fibers is more restricted and exhibits area and layer specificity.

### NA AND DA RECEPTORS ARE CO-EXPRESSED AND MEDIATE PKA ACTIVATION IN CORTICAL PYRAMIDAL NEURONS

**FIGURE 3:**
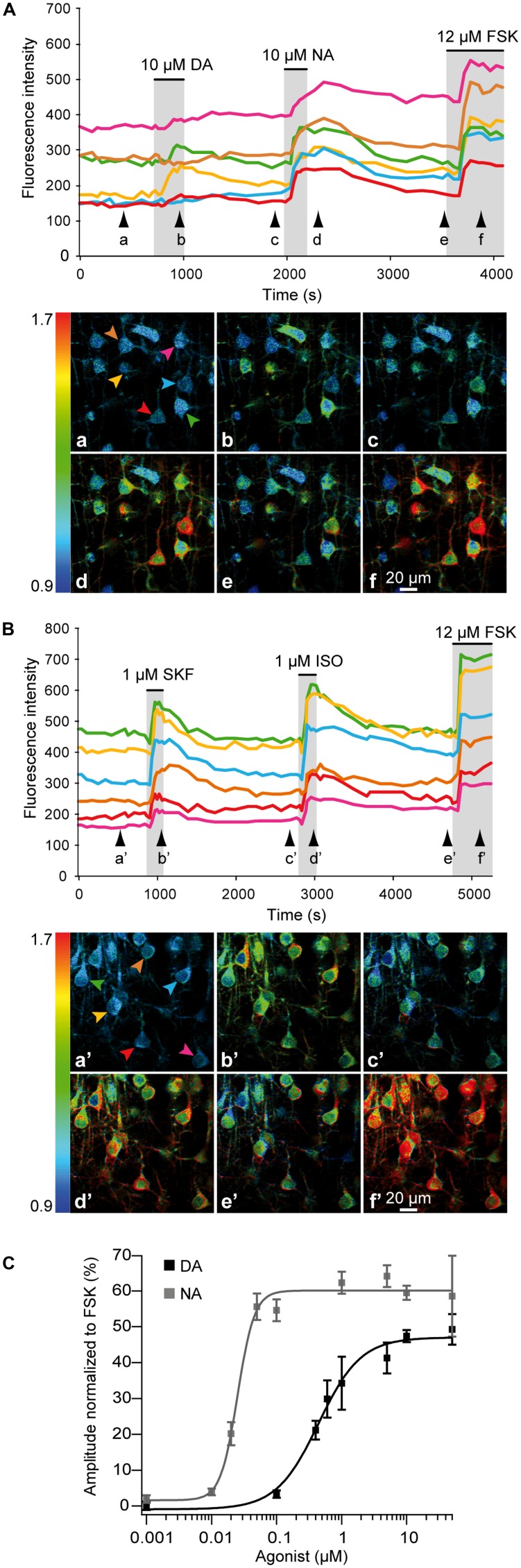
**NA and DA activate the cAMP/PKA pathway in pyramidal neurons.** 2-photon imaging of pyramidal neurons expressing the GAkdYmut fluorescent PKA sensor in parietal cortical slices. **(A)** Responses to bath application of DA, NA, and the adenylate cyclase activator FSK. Traces show the variation of fluorescence intensity measured at the soma of individual neurons indicated by arrows on pseudocolor images below. Black arrowheads indicate time points corresponding to pseudocolor images. **(B)** Responses to the D1/D5 agonist SKF38393 and the non-specific beta adrenergic agonist isoproterenol. **(C)** Dose–response curves of PKA activation by NA and DA. Each data point corresponds to the mean response measured in 10–140 individual neurons from 2 to 10 different brain slices. Curves were obtained by fitting the values with the Hill equation. NA was more potent than DA in activating PKA.

We examined responses of cortical neurons to NA, DA or specific receptor agonists in frontal, parietal, and occipital areas where DA fibers are restricted to deep layers (i.e., lateral frontal, somato-sensory, and visual cortices; see above). We used 2-photon imaging to monitor responses of layers II/III and V pyramidal neurons expressing the PKA sensor GAkdYmut (Bonnot et al., 2014) following sindbis viral transfer in acute rat brain slices (see Materials and Methods and Drobac et al., 2010; Hu et al., 2011). Response amplitudes measured at the soma were normalized to those elicited by a maximally effective concentration of the adenylate cyclase activator forskolin (FSK, 12 μM; Gervasi et al., 2007). As illustrated in **Figure 3**, successive bath application of DA (10 μM) and NA (10 μM) triggered PKA activation in most pyramidal neurons of the parietal cortex, showing that DA and NA receptors can be co-expressed in individual neurons. To estimate the proportion of responsive cells, we arbitrarily defined a threshold at 5% of the maximal fluorescence variation obtained with FSK. Using this criterion, we found that virtually all pyramidal neurons responded to NA (*n* = 45, *N* = 2) and among those, 83 ± 4% responded to DA (39 out of 45 cells). These effects were mimicked by bath application of the D1/D5 agonist SKF38393 (1 μM) or the beta adrenoceptor agonist isoproterenol (1 μM; **Figure 3**). We found that 97% of the cells (57 out of 59) responded to isoproterenol and 75% to SKF (44 out of 59), suggesting that most pyramidal neurons co-express D1/D5 and beta-adrenergic receptors. Dose–response relationships of normalized NA and DA effects showed that NA was more potent than DA in activating PKA (**Figure 3**). Indeed, the EC_50_ of NA responses was estimated at 25 ± 2 nM and that of DA at 460 ± 30 nM from the fit of their dose–response curves (see Materials and Methods). Furthermore, maximal responses to NA and DA were 60 ± 1% and 47 ± 2% of the FSK response, respectively.

### NA AND DA ACTIVATE PKA ACROSS CORTICAL REGIONS AND LAYERS

**FIGURE 4:**
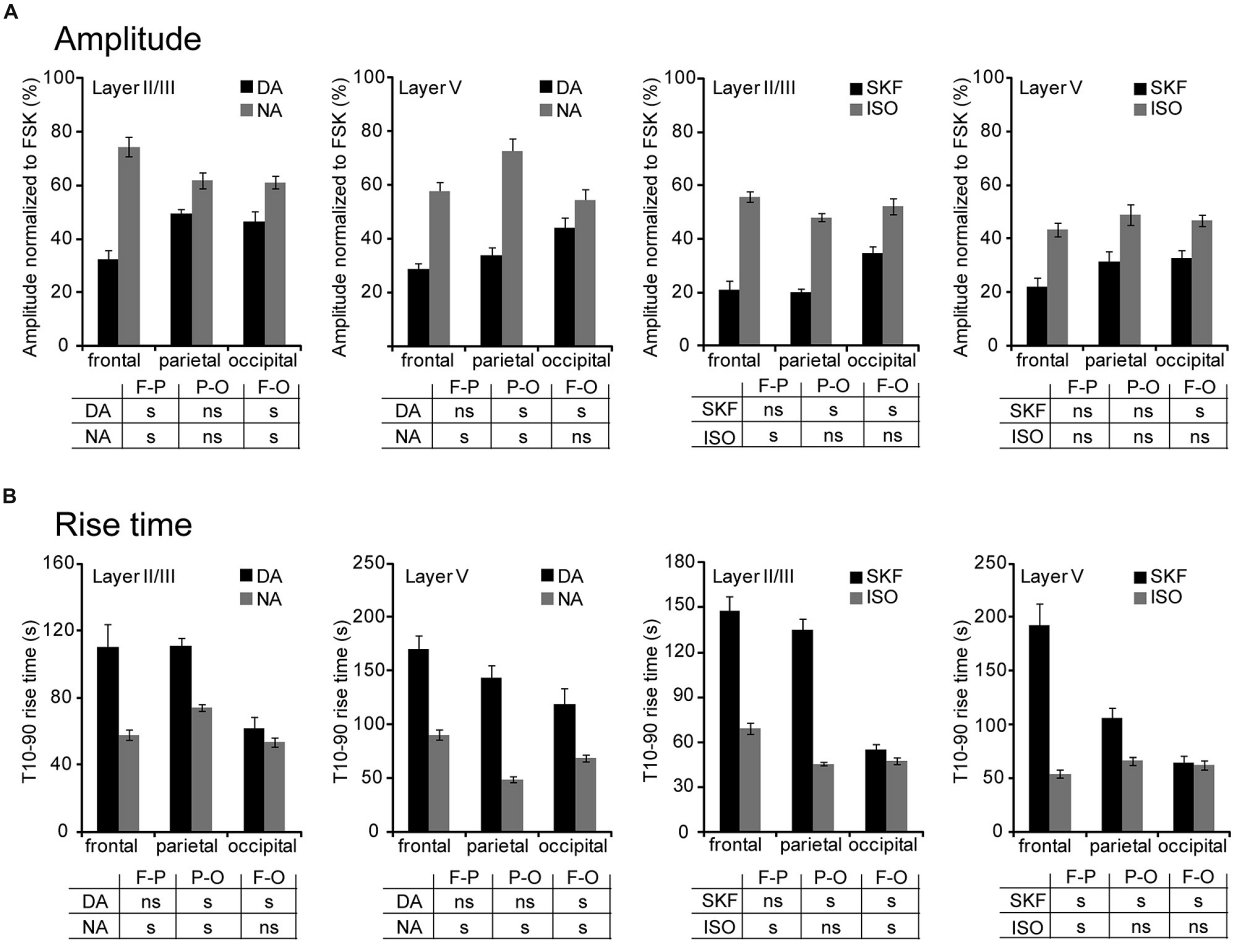
**NA and DA activate PKA across cortical regions and layers.** Data were obtained by 2-photon imaging of PKA activity in pyramidal neurons as illustrated in **Figure 3**. Imaging was performed in frontal, parietal, and occipital regions of parasagittal slices of the medio-lateral cortex that exhibits scarce DA fibers restricted to deep layers. **(A)** Amplitude of fluorescence variations normalized to the response to FSK. **(B)** Onset kinetics of responses was analyzed by calculating the 10–90% rise time. Tables indicate significant (s) or non-significant (ns) differences between values measured in the areas (F-P: frontal vs. parietal; P-O: parietal vs. occipital; F-O: frontal vs. occipital). Note the antero-posterior gradient of DA responses amplitude and velocity.

We next compared PKA activation elicited by DA, NA, SKF38393, and isoproterenol in layers II/III and V of frontal, parietal, and occipital cortices (**Figure 4** and **Table 1**). The mean amplitude of NA responses varied between 54 ± 4% in occipital layer V (*n* = 43; *N* = 4) and 75 ± 4% of the FSK effect in frontal layer II/III (*n* = 37, *N* = 2). Isoproterenol-induced responses varied similarly between 44 ± 3% in frontal layer V (*n* = 50, *N* = 4) and 56 ± 2% of the FSK effect in frontal layer II/III (*n* = 76, *N* = 4). DA and SKF38393 both caused PKA activation in all areas examined but responses were smaller than those elicited by NA or isoproterenol (**Figure 4A** and **Table 1**). DA responses were significantly smaller in both layers of the frontal cortex than in more caudal regions. Indeed, DA responses in layer II/III were 46 ± 4% in occipital (*n* = 38, *N* = 2) and 33 ± 3% of the FSK effect in the frontal cortex (*n* = 39, *N* = 3, *p* = 0.006). Similarly, DA responses in layer V were 44 ± 4% in occipital (*n* = 37, *N* = 3) and 29 ± 2% of the FSK effect in the frontal cortex (*n* = 44, *N* = 3, *p* = 0.01). Similar results were obtained with the D1/D5 agonist SKF38393, although amplitudes were smaller than those obtained with DA (**Figure 4A** and **Table 1**, *p* = 0.04 in layer II/III and *p* = 0.02 in layer V). We also observed that less than 5% of the recorded pyramidal neurons were unresponsive to NA or isoproterenol, regardless of the layer or cortical region examined. For DA, a similar result was obtained in layer II/III. However, the proportion of unresponsive cells was higher in layer V and reached 12% in the parietal cortex. For SFK38393, the proportion of unresponsive cells was above 5% in all cases with more unresponsive cells in layer V than in layer II/III. The highest value was observed in layer V of the parietal cortex with 27% of recorded neurons not responding to SFK38393. These results indicate that NA and isoproterenol induce overall larger and more homogeneous cortical responses than DA and SKF38393, whose effects increased along the antero-posterior axis.

**Table 1:** NA and DA activate PKA across cortical region and layers.

		Frontal		Parietal		Occipital	
Drugs (n/N)	Layer	Amplitude (% FSK)	T10–90% (s)	Amplitude (% FSK)	T10–90% (s)	Amplitude (% FSK)	T10–90% (s)
DA	II/III	32.5 ± 3.3 (39/3)	109.9 ± 14.1 (22/3)	49.5 ± 1.8 (242/12)	110.5 ± 4.8 (181/12)	46.4 ± 3.9 (38/2)	61.7 ± 7.1 (27/2)
	V	28.5 ± 2.4 (44/3)	170.0 ± 13.1 (30/3)	33.7 ± 3.1 (105/6)	143.4 ± 11.9 (53/6)	44.1 ± 3.8 (37/3)	119.3 ± 14.0 (37/3)
NA	II/III	74.5 ± 3.7 (37/2)	57.8 ± 3.0 (32/2)	61.9 ± 2.9 (114/7)	74.3 ± 2.0 (84/7)	61.3 ± 2.5 (77/2)	53.3 ± 2.8 (62/2)
	V	57.6 ± 3.5 (26/3)	90.2 ± 4.9 (21/3)	72.5 ± 5.0 (29/2)	48.8 ± 2.8 (26/2)	53.9 ± 3.5 (43/4)	68.4 ± 3.2 (35/4)
SKF	II/III	21.1 ± 3.4 (31/4)	147.4 ± 9.9 (39/4)	19.9 ± 1.4 (196/10)	134.9 ± 7.9 (81/10)	34.5 ± 2.6 (40/4)	55.2 ± 3.4 (31/3)
	V	22.0 ± 3.6 (36/4)	192.4 ± 20.2 (24/3)	31.4 ± 3.8 (60/4)	106.3 ± 8.9 (32/4)	32.9 ± 2.8 (69/5)	64.6 ± 5.9 (28/3)
ISO	II/III	55.8 ± 2.0 (76/4)	69.2 ± 3.5 (70/4)	48.1 ± 1.5 (172/7)	45.7 ± 1.2 (98/7)	52.3 ± 3.1 (40/3)	47.5 ± 2.5 (32/3)
	V	43.5 ± 2.5 (50/4)	54.0 ± 3.8 (46/4)	48.9 ± 3.8 (60/4)	65.9 ± 4.1 (47/4)	46.8 ± 2.1 (135/5)	62.7 ± 4.3 (50/5)

We also examined the onset kinetics of PKA responses by determining their 10–90% rise time (**Figure 4B** and **Table 1**). Kinetics of NA and isoproterenol responses exhibited moderate regional differences. Conversely, DA and SKF38393 response onsets were faster in occipital than in frontal regions with intermediate values in the parietal cortex. This was observed in both layers II/III and V (0.01 < *p* ≤ 0.003 for DA, *p* < 0.001 for SKF). As a consequence of these regional variations, the onset kinetics of DA-receptor mediated responses were markedly slower than those of NA-receptor mediated responses in the frontal cortex, but were comparable in the occipital cortex. These results confirm that NA and isoproterenol induce overall more homogeneous cortical responses and indicate that DA and SKF38393 effects increase in amplitude and velocity along the antero-posterior axis.

### CORTICAL EFFECTS OF NA AND DA INVOLVE BOTH Gs- AND Gi-COUPLED RECEPTORS

**FIGURE 5:**
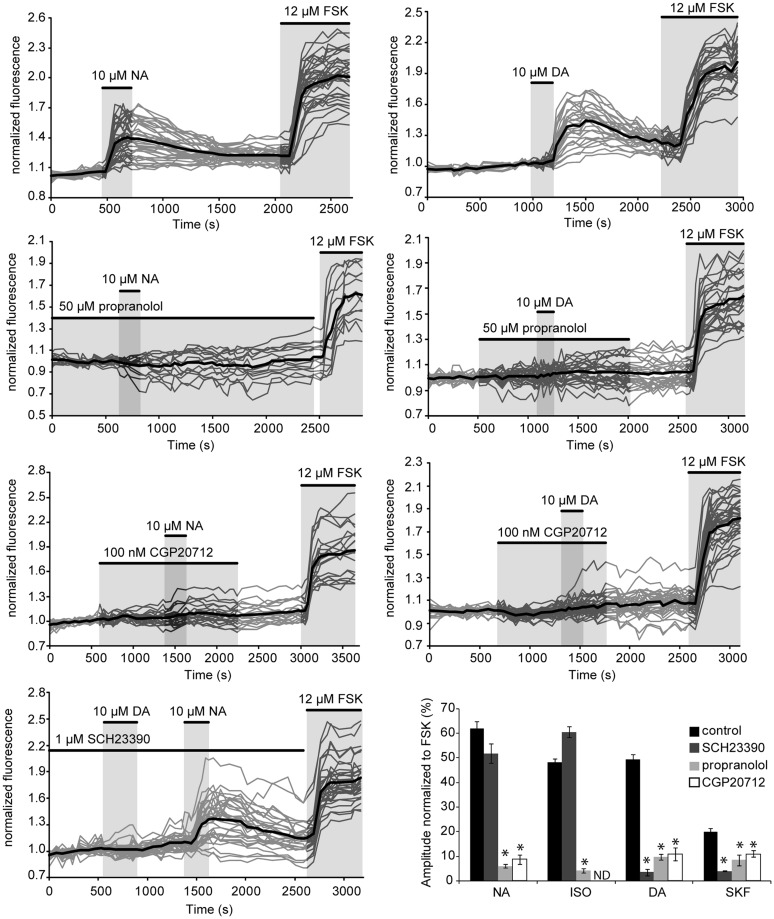
**NA and DA receptors involved in PKA activation.** Graphs show the effects of antagonists propranolol (beta adrenergic), CGP20712 (beta1-specific) and SCH23390 (D1/D5) on responses to NA and DA of layer II/III pyramidal neurons of the parietal cortex. Gray traces correspond to the fluorescence intensity normalized to the baseline value measured on individual neurons expressing the PKA sensor in parietal cortical slices (averaged traces are in black). Histograms summarize the effects of these antagonists on responses to NA, DA, the beta adrenergic agonist isoproterenol and the D1/D5 agonist SKF38393. ND: not determined; but Castro et al. (2010) reported inhibition of isoproterenol responses in cortical pyramidal neurons by CGP20712 in similar experimental conditions. Values represent the mean response of 20–200 neurons recorded in 2–12 different brain slices. Statistical significance relative to control responses is indicated by an asterisk. Note that DA responses were prevented by both beta adrenergic antagonists whereas NA responses were insensitive to the D1/D5 antagonist.

Gs-coupled NA and DA receptors involved in PKA activation were characterized in parieto-cortical layer II/III pyramidal neurons using specific antagonists (**Figure 5** and **Table 2**). The non-selective beta-adrenergic receptor antagonist propranolol (50 μM, *N* = 2, *n* = 22) and the specific beta1 antagonist CGP20712 (100 nM, *N* = 3, *n* = 40) reduced PKA activation by NA (10 μM) by more than 90% (**Figure 5**). Responses to isoproterenol (1 μM) were also reduced by propranolol to a similar extent (**Table 2**). These results indicate that NA-induced PKA activation in cortical pyramidal neurons is primarily mediated by beta1-adrenergic receptors. Responses to DA (10 μM) and to SKF38393 (1 μM) were almost abolished in the presence of the D1/D5 receptor antagonist SCH23390 (1 μM; **Table 2**). These results indicate that DA-induced PKA activation in cortical pyramidal neurons is mediated by D1/D5 receptors. NA and isoproterenol responses were not significantly altered by the D1/D5 antagonist SCH23390 (1 μM), showing that NA effects did not involve DA receptors. Surprisingly, we found that responses to DA and SKF38393 were inhibited by beta adrenergic antagonists. Indeed, propranolol and CGP20712 similarly reduced responses to DA (10 μM) by ∼80% and responses to SKF38393 (1 μM) by ∼50% (**Figure 4** and **Table 2**), suggesting that DA effects may involve beta1-D1/D5 heteromeric receptors.

**Table 2:** PKA stimulation involves beta1 adrenergic and D1/D5 dopaminergic receptors.

	NA Amplitude (% FSK)	ISO Amplitude (% FSK)	DA Amplitude (% FSK)	SKF Amplitude (% FSK)
Control (n/N)	61.9 ± 2.9 (114/7)	48.1 ± 1.5 (172/7)	49.5 ± 1.8 (242/12)	19.9 ± 1.4 (196/10)
SCH23390 (n/N)	51.8 ± 3.9 (56/2)	60.5 ± 2.3 (66/2)	3.5 ± 1.4 (56/2)	4.0 ± 0.1(49/4)
Propranolol (n/N)	6.0 ± 0.7 (22/2)	4.1 ± 0.8 (21/2)	9.7 ± 1.3 (57/2)	8.5 ± 2.1(24/2)
CGP20712 (n/N)	8.8 ± 1.9 (40/3)	ND	11.0 ± 2.7 (54/2)	11.3 ± 1.2 (101/3)

**FIGURE 6:**
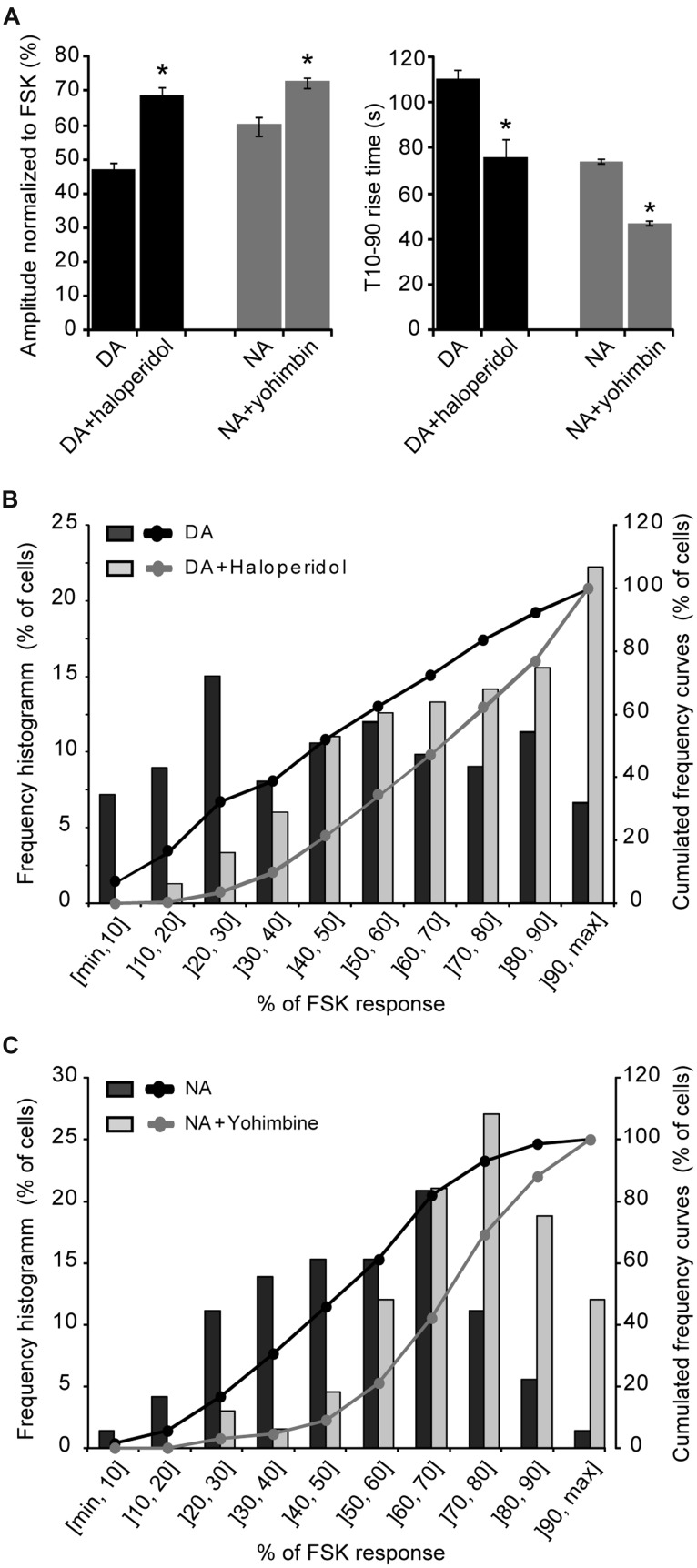
**Gi-coupled receptors dampen NA- and DA-induced PKA activation. (A)** Mean amplitude and rise time of NA- and DA-induced (10 μM each) PKA activation measured on layer II/III pyramidal neurons of the parietal cortex in the absence or presence of the D2-like antagonist haloperidol (1 μM) or the alpha2 adrenergic antagonist yohimbin (1 μM). **(B,C)** Distribution of FSK-normalized DA or NA response amplitudes measured in the same sample of neurons as in **(A)** in absence or presence of haloperidol or yohimbin. The left axis corresponds to the histogram of the proportion of cells for each amplitude interval and the right axis to the curve of cumulated frequency over the amplitude intervals. Note the shift of NA and DA response amplitude distributions toward higher amplitudes in the presence of antagonists of Gi-coupled receptors.

We next examined the contribution of Gi-coupled receptors in the response of parieto-cortical layer II/III pyramidal neurons to NA and DA (**Figure 6** and **Table 3**). Inhibition of the alpha2 adrenoceptor by yohimbin (1 μM) significantly increased the mean amplitude of the NA response by 17% (*p* = 0.007). Similarly, the D2-like receptors antagonist haloperidol (1 μM) significantly increased the DA response by 30% (*p* < 0.001). Haloperidol and yohimbin also increased the velocity of DA and NA responses. Indeed, in the presence of haloperidol, the 10–90% rise time of DA responses decreased significantly from 111 ± 5 s (*n* = 181, *N* = 12) to 76 ± 3 s (*n* = 84, *N* = 4, *p* < 0.001). Yohimbin application similarly resulted in a decreased rise time of NA responses from 74 ± 2 (*n* = 84, *N* = 7) to 47 ± 2 s (*n* = 119, *N* = 5; *p* < 0.001; **Figure 6** and **Table 3**). We previously noted a large heterogeneity in the amplitudes of DA responses (**Figures 3** and **5**). We thus examined whether antagonists of Gi-coupled receptors could modify the distribution of DA and NA response amplitudes (**Figures 6B,C**). The plot of DA response amplitudes in control condition had a broad distribution, with almost half of the cells exhibiting responses below 40% of the FSK effect, and only 6% responding above 90%. The distribution of DA response amplitudes was shifted to higher values in the presence of haloperidol, with only 10% of the cells responding below 40% of the FSK effect, and 22% responding above 90%. Although NA response amplitudes were less variable, yohimbine also markedly shifted their distribution toward higher values. These results indicate that Gi-coupled receptors are co-activated with Gs-coupled receptors by DA and NA, and significantly limit DA- and NA-induced PKA activation in cortical pyramidal neurons.

**Table 3:** NA and DA effects involve alpha2 adrenergic and D2-like dopaminergic receptors.

	DA	DA + Haloperidol	NA	NA + Yohimbin
Amplitude (% FSK) (n/N)	49.5 ± 1.8 (242/12)	69.7 ± 1.9 (143/6)	61.9 ± 2.9 (114/7)	72.3 ± 1.7 (133/6)
T10-90% (s) (n/N)	110.5 ± 4.8 (181/12)	75.9 ± 3.0 (84/4)	74.3 ± 2.0 (84/7)	46.8 ± 1.9 (119/5)

## DISCUSSION

Genetic labeling confirmed that NA neurons innervate the entire cortex whereas DA fibers were present in all layers of restricted medial and lateral cortical areas but only in deep layers of other areas. Imaging of PKA activity in frontal, parietal, and occipital regions with scarce DA fibers revealed that layers II/III and V pyramidal neurons widely respond to NA and DA. The EC50 of NA response was more than 10-fold lower than for DA. Responses to NA and isoproterenol had higher amplitude, velocity and were more homogeneous than those elicited by DA and SKF38393, whose amplitude and velocity increased along the antero-posterior axis. NA- and DA-induced PKA activation was mediated by Gs-coupled beta1 and D1/D5 receptors. Beta adrenergic antagonists inhibited both NA and DA responses whereas the effect of a D1/D5 antagonist was selective of DA responses. NA and DA responses also involved Gi-coupled alpha2 adrenergic and D2-like dopaminergic receptors that markedly reduced the amplitude and velocity of responses and contributed to their cell-to-cell heterogeneity.

### DISTRIBUTION OF CATECHOLAMINERGIC FIBERS IN THE CORTEX

The overall distribution of GFP-labeled DA and NA fibers in the cortex we describe is largely congruent with previous maps obtained using histochemistry, radiolabeling or immunohistochemical approaches (Morrison et al., 1978, 1979; Descarries et al., 1987; Berger et al., 1991; Nicholas et al., 1996; Venkatesan et al., 1996; Latsari et al., 2002; Papay et al., 2006; Paschalis et al., 2009). NA fibers were present in all layers and areas of the cortex, although their density exhibited regional and laminar variations. Conversely, DA fibers were present in all layers of two medial and lateral rostro-caudal bands comprising frontal, cingulated, rhinal cortices as well as the agranular area of the insular cortex but were confined to deep layers of other cortical areas. The present study shows that DA receptors are functionally expressed in areas with scarce or no DA fibers, consistent with the presence of their cognate mRNAs and proteins (Ariano and Sibley, 1994; Khan et al., 1998; Luedtke et al., 1999; Lemberger et al., 2007; Rivera et al., 2008; Oda et al., 2010). The source and nature of the endogenous ligand activating these DA receptors is still unclear but several studies indicate that DA can be co-released from NA fibers where it is present as the biosynthetic precursor of NA. Indeed, Devoto et al. (2005, 2008) showed that electrical stimulation of the LC results in VTA-independent NA and DA increases in the frontal cortex but also in cortices devoid of DA fibers. Similar conclusions were drawn from a study of amphetamine-induced DA release in the dorsal hippocampus, which is prevented by TH knock-down using siRNAs in the LC but not in the VTA (Smith and Greene, 2012). Hence, LC fibers are a plausible source of endogenous DA that provides a rationale for the widespread functional expression of Gs- and Gi-coupled DA receptors in the cortex.

### NA AND DA RECEPTORS WIDELY ACTIVATE cAMP/PKA IN THE CORTEX

Our results indicate that NA and DA receptors mediate activation of the cAMP/PKA pathway in pyramidal neurons throughout the cortex, even in areas with scarce or no DA fibers. Indeed, we observed robust PKA activation by NA and DA in layer II/III of fronto-lateral, parietal and occipital regions of the cortex where DA fibers are restricted to deep layers. These results indicate that NA released by LC fibers widely influences the function of neural networks in the entire cortical mantle. The same conclusion applies to DA, regardless of its source. We found that NA responses were generally larger, with faster onsets and exhibited less regional variability than DA responses. NA-induced PKA activation was mimicked by the beta agonist isoproterenol and primarily mediated by beta 1 receptors, as shown by the powerful inhibitory effect of the specific antagonist CGP20712 on NA responses. These results are consistent with the predominant expression of beta1 over beta2–3 receptors in the cortex and with the widespread expression of beta1 receptors in layers and areas of the rodent cortex (Nicholas et al., 1996; Paschalis et al., 2009). DA-induced PKA activation exhibited similar properties in layer II/III and layer V, but both the amplitude and velocity of DA responses increased from rostral to caudal regions. DA responses were mimicked by the D1/D5 agonist SKF38393 and prevented by the D1/D5 receptor antagonist SCH23390, pointing to the involvement of D1 and/or D5 receptors in DA-induced PKA activation and its rostrocaudal variations. Regional and laminar differences of D1 and D5 expression levels have been reported in the rodent cortex (Ariano and Sibley, 1994; Ciliax et al., 2000). However, the lack of specific pharmacological tools differentiating these receptors makes it difficult to assign the present functional variations to the regional levels of these receptors. The possibility that D1/D5 receptors forms heteromers with beta1 adrenoceptors further complicates the interpretation of DA response regional variations.

We found that the beta blocker propranolol and the beta1 antagonist CGP20712 inhibited NA responses. Surprisingly, these antagonists also prevented DA responses and largely reduced SKF38393 responses suggesting that DA-induced PKA activation in the cortex may involve beta1 adrenergic receptors. Previous studies established that DA effects can occur through the activation of adrenergic receptors (Aguayo and Grossie, 1994; Rey et al., 2001; Cornil et al., 2002). The present observations that DA effects were mimicked by SKF38393 and that response to these agonists were prevented by the D1/D5 antagonist SCH23390 imply a direct effect of DA through D1/D5, but not beta adrenergic receptors. DA receptors are known to form heteromers with a variety of receptors including beta adrenoceptors (Rebois et al., 2012; Fuxe et al., 2014). Moreover, cross antagonism by specific antagonist has been demonstrated for the beta1-D4 receptor heteromer (Gonzalez et al., 2012). Hence, these reports substantiate the hypothesis that D1/D5 receptors exist as heteromers with beta1 adrenoceptors in the cortex, thus exhibiting the specific pharmacological pattern we observed.

### NA AND DA CONTROL CORTICAL cAMP/PKA SIGNALING VIA Gs- and Gi-COUPLED RECEPTORS

Our results indicate that DA and NA exert a balanced control on cortical cAMP/PKA signaling by activating both Gs- and Gi-coupled receptors. We found that Gs-coupled beta1 and D1/D5 receptors are co-expressed in virtually all layers II/III and V pyramidal neurons throughout the antero-posterior axis of the cortex. The effects of yohimbin and haloperidol on NA and DA response in pyramidal neurons of parietal layer II/III suggest that alpha2 adrenoceptors and D2-like receptors are also broadly expressed in the cortex. This is in agreement with previous reports showing widespread immunoreactivity for alpha2 and D4 receptors in the cortex (Venkatesan et al., 1996; Khan et al., 1998; Rivera et al., 2008) and for D2 receptors in pyramidal cells of the medial prefrontal cortex (Zhang et al., 2010).

We showed that kinetics and amplitudes of catecholaminergic signals vary between cortical areas. It was previously shown in pyramidal neurons of the prefrontal cortex that intracellular elements of the cAMP/PKA cascade, such as phosphodiesterases, phosphatases, or the coupling between receptor and adenylate cyclases are essential in controlling the kinetics and shape of D1-like responses (Castro et al., 2013).

We found that block of alpha2 adrenoreceptors and D2-like receptors by yohimbin and haloperidol increased the amplitude and velocity of NA and DA responses. This is consistent with an inhibitory effect of these receptors on cAMP/PKA signaling that limits DA- and NA-induced PKA activation in cortical pyramidal neurons. In the case of DA, the balance of Gs- and Gi-coupled receptors effects was such that many cells were not responsive to a saturating DA concentration. Hence, regulation of the balance between Gs- and Gi-coupled receptor activities can largely influence the net effect of catecholamines on cortical neurons and networks. Interestingly, DA levels in basal conditions or upon LC stimulation are comparable to those of NA in various cortical regions, but markedly lower than DA levels in basal ganglia (Devoto et al., 2005, 2008). Our measurements of EC50 values are indicative of a ∼20-fold lower affinity of DA for D1/D5 receptors than of NA for beta1 receptors, while NA and DA Gi-coupled receptors exhibit higher affinities than their cognate Gs-coupled receptors (for a detailed comparison of pharmacological properties of receptors, see the International Union of Basic and Clinical Pharmacology database: http://www.guidetopharmacology.org/). This suggests that, at low catecholamine concentration, DA may essentially trigger cAMP/PKA inhibition whereas NA effects may be more balanced. Conversely, large catecholamine increases may be required for a significant contribution of DA to cAMP/PKA stimulation. Assuming co-release of NA and DA from LC fibers in the cortex, DA may thus expand the dynamic range of LC effects on cortical cAMP/PKA signaling.

## Conflict of Interest Statement

The authors declare that the research was conducted in the absence of any commercial or financial relationships that could be construed as a potential conflict of interest.
